# Temporal trends in physical fitness among Chinese 16- to 18-year-old adolescents between 2019 and 2025: based on data from 37,067 high school students in Jinan

**DOI:** 10.3389/fpubh.2026.1816184

**Published:** 2026-05-19

**Authors:** Li Liu, Jingtang He

**Affiliations:** 1 School of Foundational Education, University of Health and Rehabilitation Sciences, Qingdao, Shandong, China; 2 School of Physical Education, Huainan Normal University, Huainan, Anhui, China

**Keywords:** adolescents, growth and development, physical fitness, physical health, temporal trends

## Abstract

**Background:**

This study examined temporal trends in physical fitness assessed using the National Student Physical Health Standard (NSPHS) battery among 16- to 18-year-old adolescents from three senior high schools in Jinan, China from 2019 to 2025 to inform strategies to improve adolescent fitness.

**Methods:**

This study analyzed 37,067 NSPHS test records (boys: 18,966; girls: 18,101) from three senior high schools in Jinan between 2019 and 2025, representing repeated annual cross-sectional records rather than repeated follow-up of the same students, including BMI, sit and reach, 50 m sprint, 1,000 m/800 m run, pull-ups/sit-ups, and standing long jump. Year-specific means were estimated, and temporal trend plots were visually inspected to describe adjacent annual fluctuations, and sample-weighted linear regression was used to quantify trends.

**Results:**

From 2019 to 2025, height increased gradually in both boys and girls, body weight increased overall, and BMI remained largely stable. In muscular fitness, explosive strength assessed by standing long jump showed an overall decline in both sexes, upper-body muscular endurance declined in boys for pull-ups, whereas abdominal muscular endurance assessed by sit-ups improved markedly in girls. For speed and cardiorespiratory endurance, 50 m sprint time fluctuated with a slight overall decrease, while 1,000 m/800 m run time fluctuated with a slight overall increase; flexibility assessed by sit and reach remained largely stable. Small effects were observed for boys’ height, standing long jump, and pull-ups, and for girls’ height and standing long jump; a large effect was observed for girls’ sit-ups.

**Conclusion:**

From 2019 to 2025, physical fitness among 16- to 18-year-old adolescents from three senior high schools in Jinan showed largely stable BMI but mixed trends across fitness components. Performance-based indicators generally worsened (e.g., standing long jump and 1,000-m/800-m run), whereas girls showed a marked improvement in sit-ups. These findings should be interpreted as city- and school-specific rather than nationally representative, and they underscore the need for sex-specific, school-based strategies to halt declines in strength and endurance.

## Highlights

- Standing long jump and the 1,000-m/800-m run may serve as practical sentinel indicators for routine school-based monitoring within the NSPHS battery.
- The unfavorable trends in lower-limb power in both sexes and in boys’ pull-ups support sex-specific school strategies targeting strength and endurance.
- The marked improvement in girls’ sit-ups suggests that some fitness components may respond to routine school-based training and assessment-focused preparation.

## Introduction

Adolescence is a critical period for the development of physical fitness and health. Physical development during this stage has important implications not only for lifelong individual health but also for population health and the nation’s future (1). Since the beginning of the 21st century, the physical fitness of Chinese adolescents has shown periodic fluctuations and, in some domains, declines, while overweight and obesity have become increasingly prominent, likely in the context of urbanization, rising academic pressure, sedentary behavior, and changes in dietary patterns (2). In response, China has implemented the National Student Physical Fitness Standard (2014 revised edition) and the Healthy China initiative, both of which emphasize monitoring, evaluation, and feedback mechanisms (3). Regional trend analyses based on long-term, continuous physical fitness surveillance data can help identify key indicators and sensitive developmental periods, thereby providing evidence for school PE reform and targeted interventions (4). Using physical fitness test data collected from three senior high schools in Jinan between 2019 and 2025, the present study evaluates annual trends and sex differences in indicators of body morphology, physiological function, and physical fitness and aims to provide city- and school-specific evidence to inform local school-based strategies for improving adolescent physical fitness and optimizing fitness monitoring in comparable settings.

## Materials and methods

### Subjects

This retrospective, school-based study analyzed annual physical fitness test data collected from three senior high schools in Jinan, Shandong Province, China, between 2019 and 2025. The three participating schools were purposively selected based on the availability and completeness of routinely collected annual fitness records rather than by random sampling. The dataset comprised repeated annual cross-sectional records rather than repeated measurements of the same students across years, because individual identifiers were not available for longitudinal linkage. Eligible participants were students aged 16–18 years enrolled in the three participating senior high schools who completed the standardized school-administered physical fitness assessment in accordance with the National Student Physical Health Standard (NSPHS) during the study period. The three participating senior high schools were public schools and served students from both urban and rural areas. All students in the present sample were boarders. Test records were excluded if age or sex information was missing, if any key outcome variables required for the present analyses were missing, or if values were deemed implausible after data verification. The final analytic dataset comprised 37,067 test records, including 18,966 records for boys and 18,101 for girls.

### Procedures

Physical fitness data included measurements of height, weight, body mass index (BMI), sit and reach, 50-m sprint, 1,000 m (boys)/800 m (girls) run, pull-ups (boys)/1-min sit ups (girls), and standing long jump. All tests were administered in strict accordance with the 2019 National Student Physical Fitness and Health Survey and the Manual for Spot Checks and Verification of the NSPHS (5). Identical models of testing equipment were used across assessments, and quality control procedures met the required standards. All examiners received standardized training and were required to pass an assessment based on uniform operating procedures. Testing was organized by class and conducted using a station-based format, with standardized workflows and on-site supervision to ensure protocol adherence. Data were collected by designated personnel and underwent double-checking and double-entry procedures. Records with missing data, low test completion, or implausible values were excluded to ensure data quality and comparability across years. Implausible values were identified for each indicator using a mean ±3 SD rule within each sex-by-year subgroup and were rechecked before exclusion. In addition, all annual physical fitness assessments in the three participating schools were completed in November in every study year, including 2020. Although local schooling arrangements in Jinan were temporarily affected during the COVID-19 period, these disruptions did not alter the timing or core procedures of the annual November testing in the present dataset. Conducting the assessments in the same month each year helped minimize potential variation related to temperature, school schedule, and examination pressure, thereby improving comparability across years under broadly similar environmental conditions.

### Statistical analysis

Data were collected and organized using Microsoft Excel and analyzed using SPSS Statistics 26.0. Mean values of physical fitness indicators were calculated for students aged 16–18 years in the three participating schools for each study year, and temporal trend plots were generated. Because the analytic dataset consisted of repeated annual cross-sections, the analyses were designed to summarize between-year changes rather than within-student trajectories. Given the 2019–2025 observation window, the findings were interpreted as short-term annual temporal changes rather than long-term temporal trends. To address the possibility that trends were not strictly linear, adjacent year-to-year changes in the plots (e.g., 2019–2020 and 2020–2021) were visually compared, with particular attention to the period around COVID-19. To quantify linear temporal trends over the 2019–2025 study period, sample-weighted linear regression models were fitted with the annual mean physical fitness as the dependent variable and calendar year as the independent variable. Regression results are reported as unstandardized slopes (change per year) with 95% confidence intervals (CIs) and two-sided *p* values. The magnitude of change in physical fitness from 2019 to 2025 was estimated using either Cohen’s *d* or the coefficient of determination (*r*^2^), as appropriate. Effect size thresholds of 0.2 ≤ *d* < 0.5, 0.5 ≤ *d* < 0.8, and *d* ≥ 0.8 were interpreted as small, moderate, and large effects, respectively (6). When statistical tests were non-significant and effect sizes were small, the evidence for meaningful change was considered insufficient, and the significance of all tests was *p* < 0.05 (7). In interpretation, effect sizes and absolute changes in original units were prioritized over *p* values, and percentage changes were used to help judge practical relevance, given that the large sample size could render trivial differences statistically significant.

## Results

### Descriptive analysis

**Figure 1:**
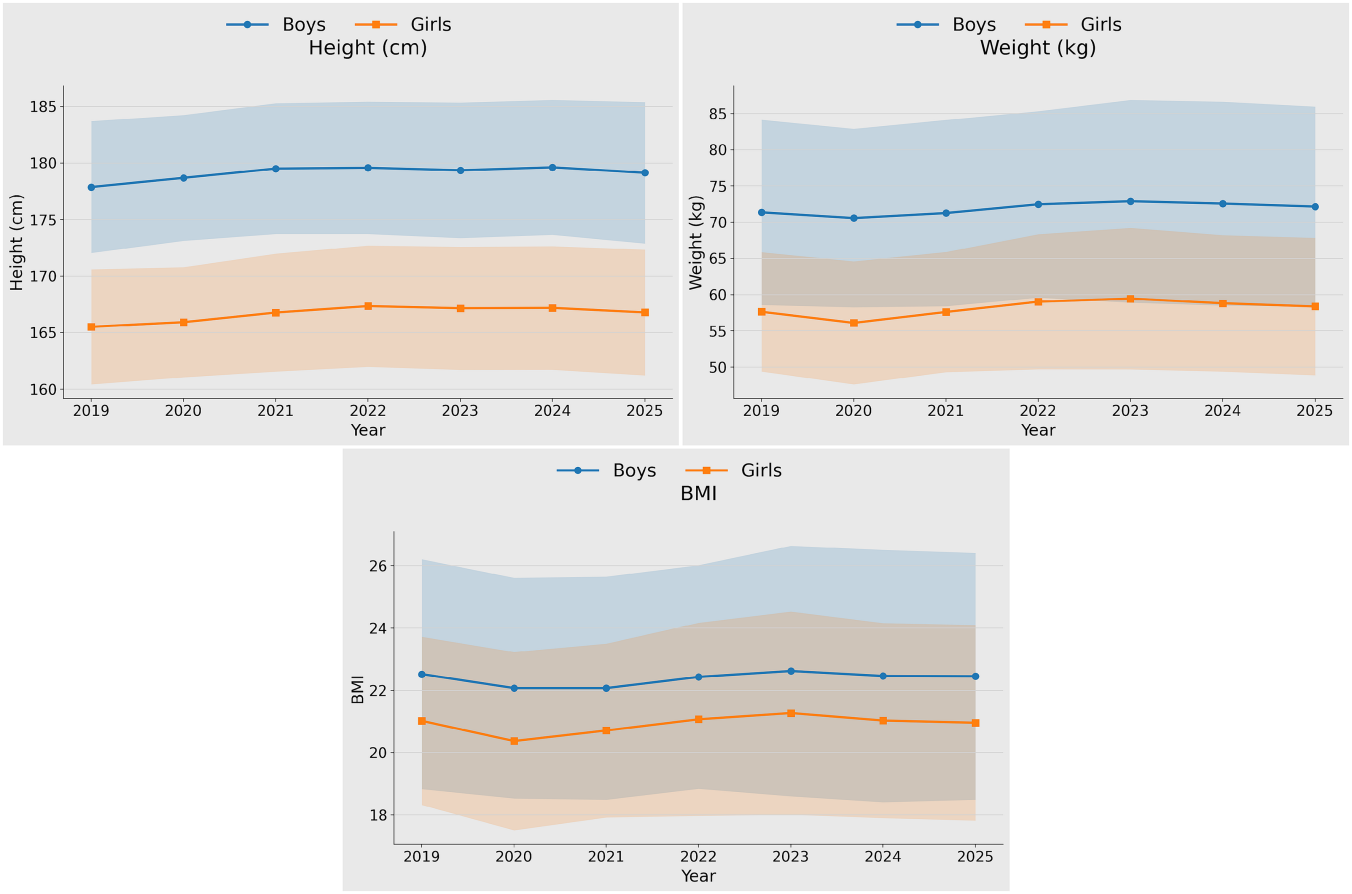
Trends in physical morphology among 16- to 18-year-old adolescents between 2019 and 2025.

From 2019 to 2025, both boys and girls aged 16–18 years in Jinan showed a gradual increase in height, while body weight increased overall. BMI remained relatively stable over the study period. Notably, both weight and BMI exhibited a transient decline around 2020, followed by a subsequent increase (Figure 1). Visual inspection of the annual plots therefore suggested short-term fluctuations around 2020 rather than a uniform year-by-year linear change for some indicators.

**Figure 2:**
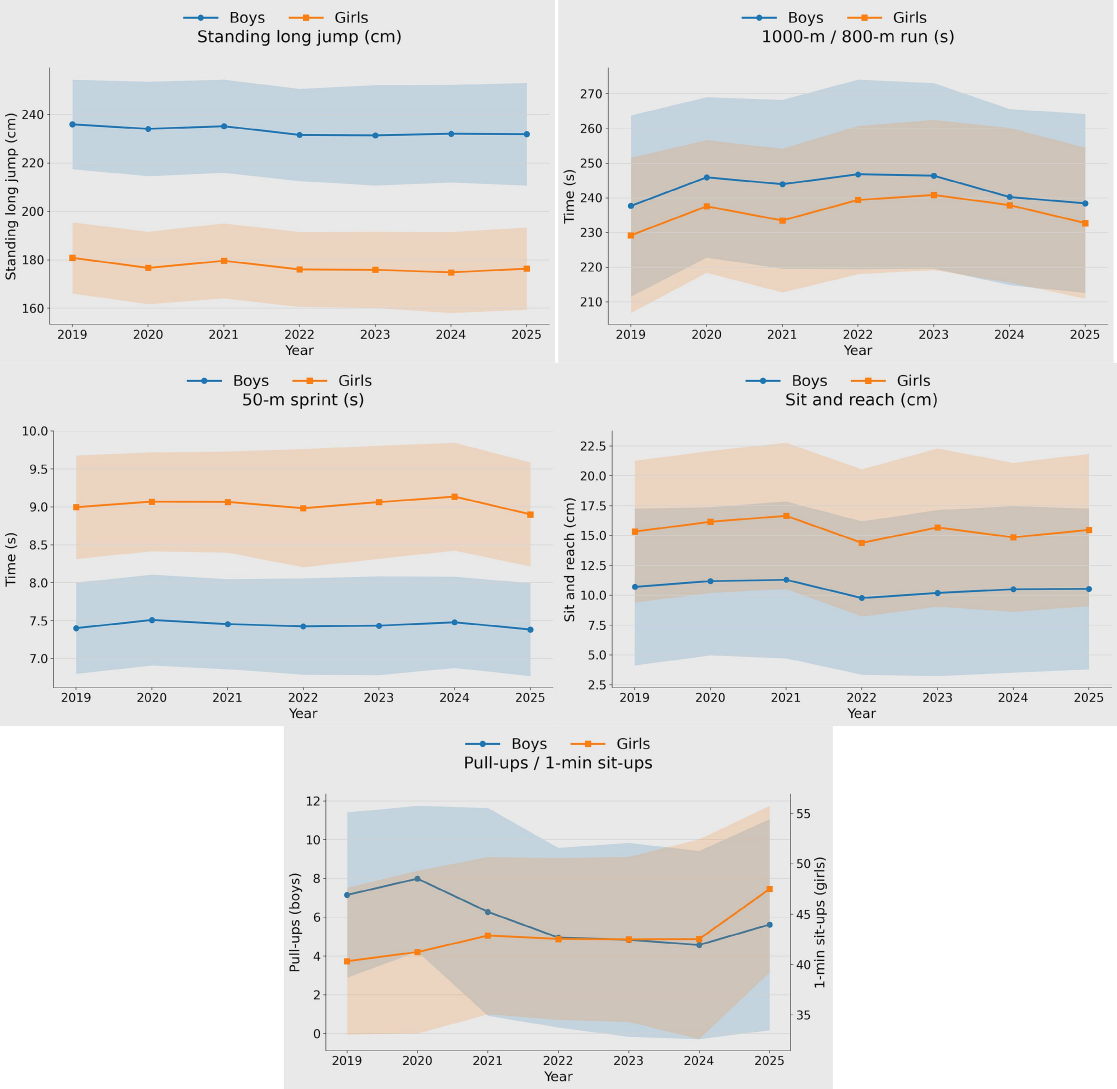
Trends in physical fitness among 16- to 18-year-old adolescents between 2019 and 2025.

Regarding muscular fitness, explosive strength assessed by standing long jump showed an overall declining trend between 2019 and 2025. Boys also demonstrated a decline in upper-body muscular endurance assessed by pull-ups, whereas girls exhibited a marked improvement in abdominal muscular endurance assessed by sit-ups (see Figure 2).

### Regression analysis

Across all physical fitness indicators, the standardized magnitude of change from 2019 to 2025 (expressed as Cohen’s *d* , SD units) ranged from 0.02 to 0.92, and the corresponding relative change ranged from −21.40 to +17.72%.

Among boys, tests of temporal trends were statistically significant (*p* < 0.05) for height, weight, BMI, standing long jump, 50-m sprint, 1,000-m run, pull-ups, and sit and reach. Small effects were observed for height, standing long jump, and pull-ups, whereas the remaining indicators showed trivial effects; no moderate or large effects were observed.

Among girls, all corresponding indicators also demonstrated statistically significant temporal trends (*p* < 0.05). A large effect was observed for sit-ups, and small effects were found for height ( *d* = 0.24) and standing long jump (see Table 1).

**Table 1:** Changes in physical fitness levels among 16- to 18-year-old adolescents from 2019 to 2025.

Index	Boys (*n* = 18,966)							Girls (*n* = 18,101)						
	${\bar{X}}_{2019}$	$\bar{X}$ _2025_	$\bar{X}$ _change_	*s*	95% *CI*	*d*	*r^2^*	${\bar{X}}_{2019}$	${\bar{X}}_{2025}$	$\bar{X}$ _change_	*s*	95% *CI*	*d*	*r^2^*
Height/cm	177.88	179.14	1.26	0.18^*^	0.133, 0.227	0.21	0.003	165.52	166.79	1.27	0.22^*^	0.182, 0.258	0.24	0.007
Weight/kg	71.37	72.16	0.79	0.26^*^	0.177, 0.343	0.06	0.002	57.64	58.37	0.73	0.32^*^	0.254, 0.386	0.08	0.005
BMI/(kg/m^2^)	22.52	22.45	−0.07	−0.04^*^	−0.058, −0.022	−0.02	0.001	21.02	20.96	−0.06	−0.06^*^	−0.088, −0.032	−0.02	0.001
Standing long jump/cm	235.92	231.88	−4.04	−0.67^*^	−0.805, −0.535	−0.20	0.005	180.83	176.39	−4.44	−0.71^*^	−0.825, −0.595	−0.28	0.008
50-m sprint/s	7.40	7.38	−0.02	−0.01^*^	−0.014, −0.006	−0.03	0.001	8.99	8.90	−0.09	−0.01^*^	−0.015, −0.005	−0.14	0.001
1000/800 m run/s	237.68	238.40	0.72	0.42^*^	0.231, 0.609	0.03	0.001	229.21	232.74	3.53	0.54^*^	0.364, 0.716	0.16	0.002
pull-ups/sit-ups	7.15	5.62	−1.53	−0.42^*^	−0.455, −0.385	−0.31	0.029	40.34	47.49	7.15	0.87^*^	0.809, 0.931	0.92	0.042
Sit and reach/cm	10.70	10.53	−0.17	−0.09^*^	−0.130, −0.050	−0.03	0.001	15.33	15.47	0.14	0.11^*^	0.059, 0.161	0.02	0.001

^*^P < 0.05; *d*: Cohen’s *d* (2019 vs. 2025).

## Discussion

This study found that from 2019 to 2025, adolescents aged 16–18 years in Jinan showed a gradual increase in height and an overall increase in body weight, whereas BMI remained largely stable, with a transient fluctuation around 2020. These trends should be interpreted as reflecting a city- and school-specific sample rather than all Chinese adolescents. This pattern is broadly consistent with evidence from Shanghai students between 2000 and 2010, which also reported overall increases in height and weight (8). Previous studies have similarly suggested that body morphology among Chinese adolescents reflects concurrent improvements in growth and development alongside a persistent burden of overweight and obesity, with continued gains in height accompanied by increasing body mass and adiposity-related risk (9). Because the dataset did not include direct measures of nutrition, health resources, or COVID-19-related lifestyle disruption, these explanations should be interpreted cautiously as plausible but speculative rather than causal; it is only possible to note that year-specific fluctuations around 2020 were observed in the descriptive trends (10). However, the magnitude of these morphological changes was small to trivial (e.g., height +1.26 to +1.27 cm; BMI −0.06 to −0.07 kg/m^2^), suggesting statistically detectable shifts rather than clearly practice-changing differences over the six-year period. Because the annual assessments were consistently conducted in November across all study years, the transient dip around 2020 is unlikely to be explained by differences in test month or seasonal timing. A cautious interpretation is that some short-term COVID-associated behavioral changes may plausibly have contributed to these fluctuations, but the observed changes were small and may also reflect normal year-to-year variability rather than a distinct pandemic-specific effect. An important contribution of the present study is that it extends school-based trend evidence into late adolescence (16–18 years) with annual observations through 2025, thereby covering the COVID era within a relatively understudied stage of secondary school development.

In terms of muscular fitness, this study found an overall decline in indicators related to explosive power and upper-body strength, with standing long jump decreasing in both sexes and pull-ups declining in boys, whereas girls’ sit-ups improved markedly, indicating clear sex-specific divergence. This pattern is consistent with broader evidence suggesting that lower-limb explosive strength (often assessed by standing broad jump/standing long jump) has been particularly prone to deterioration since the early 2000s, with declines observed in many countries and in some periods becoming more pronounced (11). Contemporary national data also indicate that explosive strength remains challenging to improve at the population level, while some markers of muscular strength may show stabilization or modest improvement, alongside widening disparities between low and high performers (12). Because sedentary behavior, physical activity opportunities, and academic burden were not directly measured in the present dataset, these factors can only be discussed as plausible but untested explanations for the observed muscular fitness trends. Moreover, the opposite directions observed for boys’ pull-ups and girls’ sit-ups may partly reflect test-specific characteristics and school practice emphasis: pull-ups are highly sensitive to relative strength and body-mass management, making performance more vulnerable to concurrent weight gain and insufficient targeted training, whereas sit-ups may be more readily improved through routine PE-class practice and assessment-oriented preparation.

From a practical standpoint, the approximately 4-cm decline in standing long jump and the 1.53-repetition decline in boys’ pull-ups may warrant school-level attention, whereas the large improvement in girls’ sit-ups appears more likely to reflect a meaningful change in abdominal muscular endurance. The sex-specific divergence observed here, especially the combination of declining standing long jump in both sexes, declining pull-ups in boys, and markedly improving sit-ups in girls, adds nuance to the literature by showing that different components of muscular fitness may move in different directions within the same population and time period rather than changing uniformly. In relation to the international evidence synthesized by Tomkinson et al., which reported a negligible pooled long-term change in standing broad jump across 1960–2017 but declines in many countries since 2000, the approximately 4-cm decline observed over only 6 years in our sample appears directionally consistent with recent global concerns and may be somewhat steeper in absolute terms, although this comparison should be interpreted cautiously because our estimate reflects a short recent interval within a single city rather than a pooled international series (11).

In this study, time-based endurance tests fluctuated but showed a slight overall increase, and flexibility performance improved modestly. National trend analyses have similarly reported overall improvements in speed after 2010 and continued gains in flexibility among girls, whereas endurance has predominantly shown fluctuating declines, suggesting that changes across fitness components are not synchronized. Monitoring studies from regions such as Yunnan also indicate that flexibility is more likely to improve, while explosive power and some other components remain suboptimal (13). The slight improvement in sprint time and worsening of endurance performance in our sample should not be attributed to specific school PE practices or extracurricular exercise patterns, as such determinants were not directly measured in the dataset (14). By contrast, changes such as 0.02–0.09 s in 50-m sprint time or 0.14 cm in sit and reach are unlikely, on their own, to materially alter school-based fitness classification or intervention decisions, even when statistically detectable.

From a practice and policy perspective, these results suggest several concrete actions. First, because standing long jump declined in both sexes, PE curricula may need greater emphasis on lower-limb power development through progressive jump-landing tasks, plyometric drills, and neuromuscular training. Second, the worsening of boys’ pull-ups and the slight deterioration in the 1,000-m/800-m run indicate that structured, school-based strength and endurance programmes may be more appropriate than relying mainly on short-term test preparation. Third, within the NSPHS framework, standing long jump and the endurance run may be considered practical sentinel indicators for routine monitoring in late adolescence, because both are easy to administer and showed unfavorable trends in the present sample. Although the Chinese standard provides score-based performance classifications, the present secondary dataset was analyzed at the level of annual means; therefore, we could not determine whether the proportion of students below recommended fitness levels changed over time. Future surveillance should complement mean-based trend analyses with category-based reporting to better support school decision-making and policy refinement.

This study has several limitations. First, because the dataset was derived from only three senior high schools in one city and lacked detailed school-level contextual descriptors, the external validity of the findings is limited and sample representativeness beyond the participating schools cannot be fully determined. Second, because the de-identified dataset represented repeated annual cross-sections and did not retain student-level linkage or school identifiers, we were unable to examine within-student trajectories or fit multilevel models to account for potential school-level clustering, and apart from calendar year and the general information that the participating schools were public schools serving students from both urban and rural areas, no further contextual proxy variables were available for exploratory modelling. Third, the dataset did not include direct measures of sedentary behavior, academic burden, physical activity participation, or COVID-19-related school disruption; therefore, the explanatory factors discussed above should be interpreted as plausible but speculative rather than causal. Finally, although standardized protocols, examiner training, identical equipment models, and data verification procedures were used, residual measurement error related to routine school-based field testing and secondary data processing cannot be entirely excluded. Future research should consider additional determinants of adolescent physical fitness, including competitive athlete status and broader lifestyle-related factors (e.g., habitual physical activity, sedentary behavior, sleep, and dietary patterns), as well as social determinants such as family income, parental education, and commuting mode, to better elucidate factors shaping fitness trajectories and to inform evidence-based, school- and policy-level interventions.

## Conclusion

Between 2019 and 2025, physical fitness assessed using the NSPHS battery among 16- to 18-year-old adolescents from three senior high schools in Jinan showed modest improvements in body morphology but divergent, sex-specific trends across fitness components. Performance-based indicators declined overall (standing long jump, boys’ pull-ups, and 1,000-m/800-m run), whereas girls showed a substantial improvement in sit-ups. These city- and school-specific findings support sex-specific monitoring and targeted school-based strategies to curb declines in strength and endurance. In practical terms, these findings support sex-sensitive school strategies that strengthen lower-limb power-oriented activities, structured endurance training, and closer attention to standing long jump and endurance run within routine monitoring.

## Data Availability

The raw data supporting the conclusions of this article will be made available by the authors, without undue reservation.
